# The end of long-term ecological data?

**DOI:** 10.1371/journal.pbio.3003102

**Published:** 2025-04-03

**Authors:** Daniel T. Blumstein

**Affiliations:** 1 Department of Ecology and Evolutionary Biology, University of California, Los Angeles, California, United States of America; 2 The Rocky Mountain Biological Laboratory, Crested Butte, Colorado, United States of America

## Abstract

Can we ever have too much ecological field data? Are data sharing norms and the environmental costs of travel disincentivizing its collection? This Perspective advocates for proper funding and resources to enable collection of long-term ecological data, which is essential to assess animal behavior and adaptation in the face of anthropogenic change.

## Teaser

Can we ever have too much ecological field data? Are data-sharing norms and the environmental costs of travel disincentivizing its collection? Allocating proper funding and resources to the collection of long-term ecological data is essential for studies of behavior and adaptation, which are particularly important in the face of anthropogenic change.

Making scientific data accessible is a great thing for society. When granting agencies mandate public sharing of data and codes, others can re-analyze and synthesize data. Accessible data helps democratize knowledge creation because people without access to the field or the lab may still have excellent ideas and existing data can be used to address them. Accessible datasets can be combined in novel ways and answer novel questions; however, ecological field data requires an understanding of the system to sensibly analyze a dataset, and this has led some to question its use without meaningful collaboration [1].

Ecological field studies that exceed a decade or so are especially valuable because most field studies last 2–3 years – the amount of time it takes a graduate student to collect data for their thesis or dissertation. Why are new data needed? Because ecological systems are complex and because the world is changing rapidly. Indeed, it’s only through the growing collection of long-term studies [2] that we can understand whether and how populations have or can adapt to environmental change [3] and whether there are mismatches between key demographic and life history events and vegetation phenology [4]. These questions are of increasing importance in the Anthropocene and addressing fundamental questions about the limits of plasticity that are required to respond to environmental change.

Long-term ecological field studies raise another problem—the people collecting the data are also analysing it and there have been cases where those data were published by others before the people collecting the data had time to finalize their analyses and papers [1]. To address this concern, Evans [5] analyzed the subsequent use of long-term datasets that were archived in Dryad and wrote some custodians of long-term data. His conclusion was that virtually none of the datasets he examined were reused and concluded that researchers were unrealistically concerned. Nevertheless, getting scooped by someone else using a researcher’s own data before they could use it has happened, and this creates a clear disincentive to collect long-term data and share it broadly despite its importance to society.

Long-term data collection requires long-term support and the lack of ongoing support is recognized as its greatest challenge [3,6]. A recent analysis reported that nearly half of the long-term studies of mammals (191/411) had been terminated [3]. Some long-term research is supported by programmatic grants that support integrative research at a particular site (e.g., US National Science Foundation Long Term Ecological Research sites), but some funding agencies have terminated long-term projects for a variety of reasons, including changed funding priorities and lack of productivity [7]. Other long-term research emerges from one or a few researchers studying a particular system over time. Many of these projects must reinvent themselves each grant cycle to ensure ongoing funding that supports a core set of data (often population size, demographic rates, phenology, etc.). Such projects often do not have a single form of government support and may be supported by a variety of sources, including private, foundation, state, educational, and often even personal funds. Recently, a close collaborator of mine was told by our program officer while discussing our rejected NSF Long Term Research in Environmental Biology proposal that “we will never be funded” by their program “because [for the questions we proposed] the panel views us as having too much data.” While it is true we were completing our 63rd year of continuous study of yellow-bellied marmots (*Marmota flaviventer*), we have been productive [8,9]. We have worked hard to use the system for both high-quality education (many undergraduates have been first authors on papers resulting from their summer work) and outreach to the media. We attempted to make the case that the questions we developed for the proposal (how selection on age and sex vary and its consequences for understanding natural selection and evolution in the wild), which cannot be asked in short-term studies, require 10 more years of data to have a sufficiently powerful dataset. Yet, the belief that we collectively have “too much data” is not just restricted to our marmot study; some have suggested we no longer need to collect any new ecological data and our primary goal should be data synthesis.

In an extremely provocative and stimulating piece in *Nature Ecology and Evolution*, Dupont and colleagues [10] argued that ecologists, who know much better than the average person about the real consequences of climate change and habitat destruction on biodiversity loss, are not doing enough, personally, to stop it. The authors were spot-on in identifying a knowledge-action gap problem. However, some of their suggestions to address this problem will have long-term negative implications for our ability to understand and manage the world around us. Among other things, they argued that we don’t need to travel to collect more data and indeed it would be better for the Earth to simply stop collecting data and conduct metascience.

Metascience, studies that aggregate existing data to conduct comparative analyses, systematic reviews, and metanalyses are extremely important ways that we understand the world around us. Indeed, given the shortcomings of any single study, one can argue that they are essential if we wish to develop a comprehensive understanding of life on Earth. But as data collection techniques evolve, cobbling together studies may not provide the desired clarity. Indeed, a set of long-term studies may be just what is needed to better understand patterns and variation because each study (presumably) was fastidious in how data were collected. Data synthesis collaborations between long-term scientists are starting to reveal important patterns in how populations respond to anthropogenic changes (e.g., [11]).

These issues raise an interesting question: are we heading towards the end of long-term ecological data?

From a purely economic perspective, something that is free has no value and we have to be clear to incentivize data collection. By analogy, why bake a loaf of bread if you are required to give it away? With respect to publicly funded long-term research, the more apt metaphor might be “bake your loaf, eat your bit, and share the rest.” Researchers are supported by funds to collect data and have the first crack at analysis, but then are expected to share it with the scientific community. This only works if we maintain incentives to collect data, and this can be achieved by providing ongoing funding to worthy projects.

Depending upon where the studies occur, the opportunity costs of collecting long-term ecological data might be a bit different from those associated with laboratory research. Some long-term studies are on campuses [12], but many require travel to off-campus field sites [13], where fieldwork can be physically challenging, and researchers may put the rest of their lives on pause while in the field. By making ecological data freely available (a good thing for society), we disincentivize the hard work and sacrifices required to collect it, and this may, ultimately, lead to the end of newly collected data (a bad thing for society). Developing schemes to ensure ongoing support for long-term studies [6] can balance out opportunity costs.

The loss of ecological field data would also be devastating because this means that researchers are not in the field. Others have focused on the extinction of field experiences among ecologists [14] and the loss of an understanding and an appreciation of nature and the natural processes that follows.

The end of data, particularly long-term ecological data, has broad societal costs. We should support the ongoing collection of field data and we should support productive and promising long-term studies that give us the tools to understand and manage life in an increasingly dynamic world.

## References

[1] Mills JA, Teplitsky C, Arroyo B, Charmantier A, Becker PH, Birkhead TR, et al. Archiving primary data: solutions for long-term studies. Trends Ecol Evol. 2015;30(10):581–9. doi: 10.1016/j.tree.2015.07.006 26411615

[2] Clutton-Brock T, Sheldon BC. Individuals and populations: the role of long-term, individual-based studies of animals in ecology and evolutionary biology. Trends Ecol Evol. 2010;25(10):562–73. doi: 10.1016/j.tree.2010.08.002 20828863

[3] Schradin C, Hayes L. A synopsis of long-term field studies of mammals: achievements, future directions, and some advice. J Mammal. 2017;98(3):670–7.

[4] Plard F, Gaillard J-M, Coulson T, Hewison AJM, Delorme D, Warnant C, et al. Mismatch between birth date and vegetation phenology slows the demography of roe deer. PLoS Biol. 2014;12(4):e1001828. doi: 10.1371/journal.pbio.1001828 24690936 PMC3972086

[5] Evans SR. Gauging the purported costs of public data archiving for long-term population studies. PLoS Biol. 2016;14(4):e1002432. doi: 10.1371/journal.pbio.1002432 27058254 PMC4825988

[6] Lindenmayer D, Likens G, Andersen A. Value of long-term ecological studies. Austral Ecol. 2012;37(7):745–57.

[7] Jones J, Nelson MP. Long-term dynamics of the LTER Program: Evolving definitions and composition. In: Waide RB, Kingland SE, editors. The challenges of long term ecological research: a historical analysis. Gland: Springer; 2021.

[8] Blumstein DT. Yellow-bellied marmots: insights from an emergent view of sociality. Philos Trans R Soc Lond B Biol Sci. 2013;368(1618):20120349. doi: 10.1098/rstb.2012.0349 23569297 PMC3638452

[9] Armitage KB. Marmot biology: sociality, individual fitness, and population dynamics. Cambridge: Cambridge University Press; 2014.

[10] Dupont L, Jacob S, Philippe H. Scientist engagement and the knowledge-action gap. Nat Ecol Evol. 2025;9(1):23–33. doi: 10.1038/s41559-024-02535-0 39304789

[11] Van de Walle J, Fay R, Gaillard J-M, Pelletier F, Hamel S, Gamelon M, et al. Individual life histories: neither slow nor fast, just diverse. Proc Biol Sci. 2023;290(2002):20230511. doi: 10.1098/rspb.2023.0511 37403509 PMC10320331

[12] Savill P, Perrins C, Kirby K, editors. Wytham Woods: Oxford’s ecological laboratory. Oxford: Oxford University Press; 2010.

[13] Billick I, Price MV, editors. The ecology of place: contributions of place-based research to ecological understanding. Chicago: University of Chicago Press; 2010.

[14] Soga M, Gaston K. Extinction of experience among ecologists. Trends Ecol Evol. 2025. 10.1016/j.tree.2024.12.010 39794266

